# Anaerobic Codigestion of Municipal Wastewater, Landfill Leachate, and Crude Glycerin: Process Stability and Methane Yield Assessment Using a Screening Design

**DOI:** 10.1002/wer.70285

**Published:** 2026-02-02

**Authors:** Gustavo Henrique Pedroso, Jackeline Tatiane Gotardo

**Affiliations:** ^1^ Postgraduate Program in Agricultural Engineering (UNIOESTE/CASCAVEL/CCET/PGEAGRI), Science Technology Center Western Paraná State University (UNIOESTE) Cascavel Paraná Brazil

**Keywords:** biogas yield, organic matter removal, Plackett and Burman

## Abstract

Water resource recovery facilities often receive landfill leachate (LL), which can disrupt biological processes due to its toxicity and low biodegradability. This study evaluates the anaerobic codigestion (AcoD) of municipal wastewater (MWW), LL, and crude glycerin (CG) as a strategy to enhance organic matter removal and methane yield. Batch reactors were operated under varying conditions defined by a Plackett–Burman screening design, and methane production kinetics were modeled using modified Gompertz and Cone equations. Soluble chemical oxygen demand (sCOD) removal ranged from 67.4% to 94.3%, whereas methane yield varied between 0.076 and 0.349 L _NCH4_/g tCOD_add_ (liters of normalized methane per gram of total COD added). The highest yield was achieved with 2% LL and 1% CG, approaching the theoretical maximum. Statistical analysis revealed that increasing CG content reduced methane yield, and extending the digestion time to 40 days offered limited performance gains. Despite the presence of inhibitory compounds, most conditions showed stable digestion, with short latency phases and effective microbial adaptation. These findings demonstrate the feasibility of codigesting MWW, LL, and CG, especially under optimized proportions, and highlight the potential for energy recovery in wastewater treatment plants using biodiesel by‐products.

## Summary

- Leachate codigestion feasibility: Anaerobic codigestion of LL with MWW and CG is viable.
- CG impact: The addition of CG, a biodegradable carbon source, reduced methane yield, suggesting an optimal dosing threshold.
- Microbial adaptation: The inoculum adapted well to the substrate, with short lag phases estimated by modified Gompertz, indicating robust biological activity despite leachate toxicity concerns.
- Methane yield kinetics: Reverse L‐shaped curves indicated rapid initial methane production, likely due to glycerol. Elongated S‐shaped curves suggested slower hydrolysis, linked to lower CG content and potentially more complex substrates.

## Graphical abstract

Anaerobic codigestion of municipal wastewater with landfill leachate and crude glycerin can achieve near‐theoretical methane yields (0.349 L _NCH4_/g tCOD_add_). An optimal mixture of 2% leachate and 1% glycerin was identified, as higher glycerin content negatively impacted performance, highlighting the criticality of substrate balance for energy recovery.

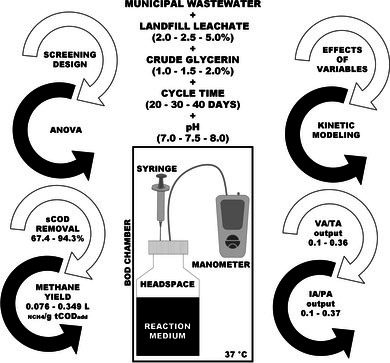

## 1 Introduction

A common approach for the treatment of landfill leachate (LL) involves its cotreatment with municipal wastewater (MWW) in an anaerobic environment (Tawfik and Tyagi 2023). LL is rich in organic matter and, when used as a cosubstrate, can enhance the potential for methane production. However, its high nitrogen concentrations pose a challenge for maintaining an optimal carbon‐to‐nitrogen (C/N) ratio (Yang et al. 2025; Zamrisham et al. 2023).

MWW, which is 99.9% water, contains substances that allow for its use beyond conventional treatment, such as energy generation processes. To achieve this, it is necessary to develop methods that aim at reducing environmental impact, which is accentuated by the water and energy crisis, making MWW a resource (Khatri et al. 2021; Zawadzki et al. 2023).

Studies show that LL is often treated in water resource recovery facilities due to their dilution capacity. However, this practice introduces challenges that require pretreatment to meet discharge standards (Kumar et al. 2023).

The problems posed by leachate are associated with its diverse chemical composition. It frequently contains heavy metals, organic contaminants, inorganic chemicals, and dissolved solids. Furthermore, recent research has reported the presence of harmful compounds such as per‐ and polyfluoroalkyl substances (PFAS) (Bouaouda et al. 2023; Gokgoz et al. 2023).

Leachate composition varies depending on the landfill's age and degradation phase. The phases are distinct because of the anaerobic processes that occur in the landfill itself, passing from younger and more biodegradable phases to older phases where the organic matter already presents recalcitrant characteristics and the predominant form of nitrogen is ammonia (Anna Tałałaj et al. 2021; Bouaouda et al. 2023).

At a global level, there is no consolidated percentage regarding the cotreatment of LL and MWW. However, countries such as the United States and Poland commonly direct this effluent to their water resource recovery facilities, leveraging existing infrastructure. In contrast, countries like France and Ireland exhibit low adherence to treatment facilities that accept LL (Brennan et al. 2017; Gokgoz et al. 2023; Patel et al. 2021).

In Brazil, 56.0% of the population has access to sanitation services, according to data from the 2023 National Sanitation Information System (SNIS), revealing that the volume of MWW treated during this period amounted to 4956.6 million m^3^ per year (Secretaria Nacional de Saneamento Ambiental 2023). Of the existing treatment plants, 37% employ biological treatment utilizing an Upflow Anaerobic Sludge Blanket (UASB) reactor with or without subsequent posttreatment (von Sperling 2016).

The application of biological methods for the treatment of MWW is associated with its high biodegradability; however, its composition varies based on the living standards of the population served, climatic conditions, and other environmental factors (Vrsalović et al. 2023). Additionally, a significant aspect is the reduced demand for chemicals and energy in these processes, which ensures efficiency in the removal of nutrients and organic matter (Ya'acob et al. 2022).

New research has sought to develop treatment methods for MWW or improve those that already exist, as is the case with combined treatments, which are used to increase efficiency or achieve synergistic advantages offered by the mixtures (Cengiz et al. 2024; Vrsalović et al. 2023; Ya'acob et al. 2022). In this sense, aspects that affect the viability of treatment need to be considered, such as the high organic load of some wastewater and the seasonal impact of those dependent on climatic conditions (Johnson and Mehrvar 2023).

In the case of LL being a complex matrix with a low C/N ratio, Lanzetta et al. (2021) needed to add a carbon source to the mixture used in their research to treat the old leachate through biological nitrogen removal processes. Recent research, such as that by Haidari et al. (2023) and Johnson and Mehrvar (2023), studied techniques that would enable the treatment of leachate from landfills by integrating processes.

In mixtures, the addition of leachate as a cosubstrate provides a supplement of macro‐ and micronutrients that the wastewater under treatment needs, and in cases where the process used is anaerobic, the production of clean energy through the generation of methane and hydrogen is possible (Saranga et al. 2020).

In addition, an effluent that can be used as a carbon source is crude glycerin (CG), which is a by‐product of biodiesel production already used in laboratory‐scale biological treatment of LL, where it is possible to achieve methane yields of 0.3 to 0.42 L _NCH4_/g COD, as shown by research by Takeda et al. (2022) and De Castro et al. (2021).

Its primary source of biodegradable carbon is glycerol, which can be generated during the anaerobic digestion of lipids, particularly in the hydrolysis phase (Li et al. 2022a). As such, glycerol is a compound that can be readily assimilated by microorganisms, contrasting with LL from aged landfills, which often contains recalcitrant or poorly biodegradable organic matter (Jamshidinasirmahale et al. 2024).

Thus, the use of CG in this process would offer, in addition to the treatment advantage previously mentioned, an economic benefit, as its production surplus could be utilized beyond its conventional applications (Bansod et al. 2025; Tu et al. 2017). In the Brazilian context, presenting uses for this effluent is relevant since the country has policies that encourage the production and use of biodiesel, favoring the generation of CG (Grangeia et al. 2022).

Considering the limited availability of studies that have simultaneously employed all three substrates, the main objective of this study is to conduct a preliminary assessment of the methane yield using the anaerobic codigestion (AcoD) process combining MWW, LL, and CG, along with verifying its treatability. For this purpose, the screening design proposed by Plackett and Burman was used to reduce the number of experiments, verify the effects of the selected variables on the obtained responses, and determine their significance in the process (Braga et al. 2018; Caroca et al. 2021; Sha et al. 2019).

## 2 Materials and Methods

### 2.1 Sample Collection and Experiment Setup

The MWW was obtained from a public sanitation service provider located in the municipality of Cascavel, Paraná, in southern Brazil, that carries out treatment using a UASB reactor followed by an optional lagoon. Twenty liters of MWW was collected after the Parshall flume of preliminary treatment only once. Ten liters of untreated LL was collected in a landfill in the same municipality that uses a system of lagoons to treat this effluent. The Sustainable Technologies Laboratory (LABTES) provided the CG samples for this study. This laboratory is located on the premises of the Western Paraná State University, Cascavel Campus, and produces biodiesel from vegetable oil.

The sludge used as reactor inoculum was obtained from a UASB reactor and characterized for its presence of total, fixed and volatile solids, fixed suspended, and volatile suspended. The tests were conducted in batch reactors with a total volume of 1.0 L, with 0.225 L of sample, 0.750 L of headspace, and 0.025 L of support material of the model MBBRing 681 26 8 LIGHT used to enhance microbial adhesion and biofilm development. The headspace of 75% was chosen to ensure sufficient biogas accumulation capacity between daily measurements, minimizing overpressure in the closed system and preventing leaks through the rubber septa.

**FIGURE 1:**
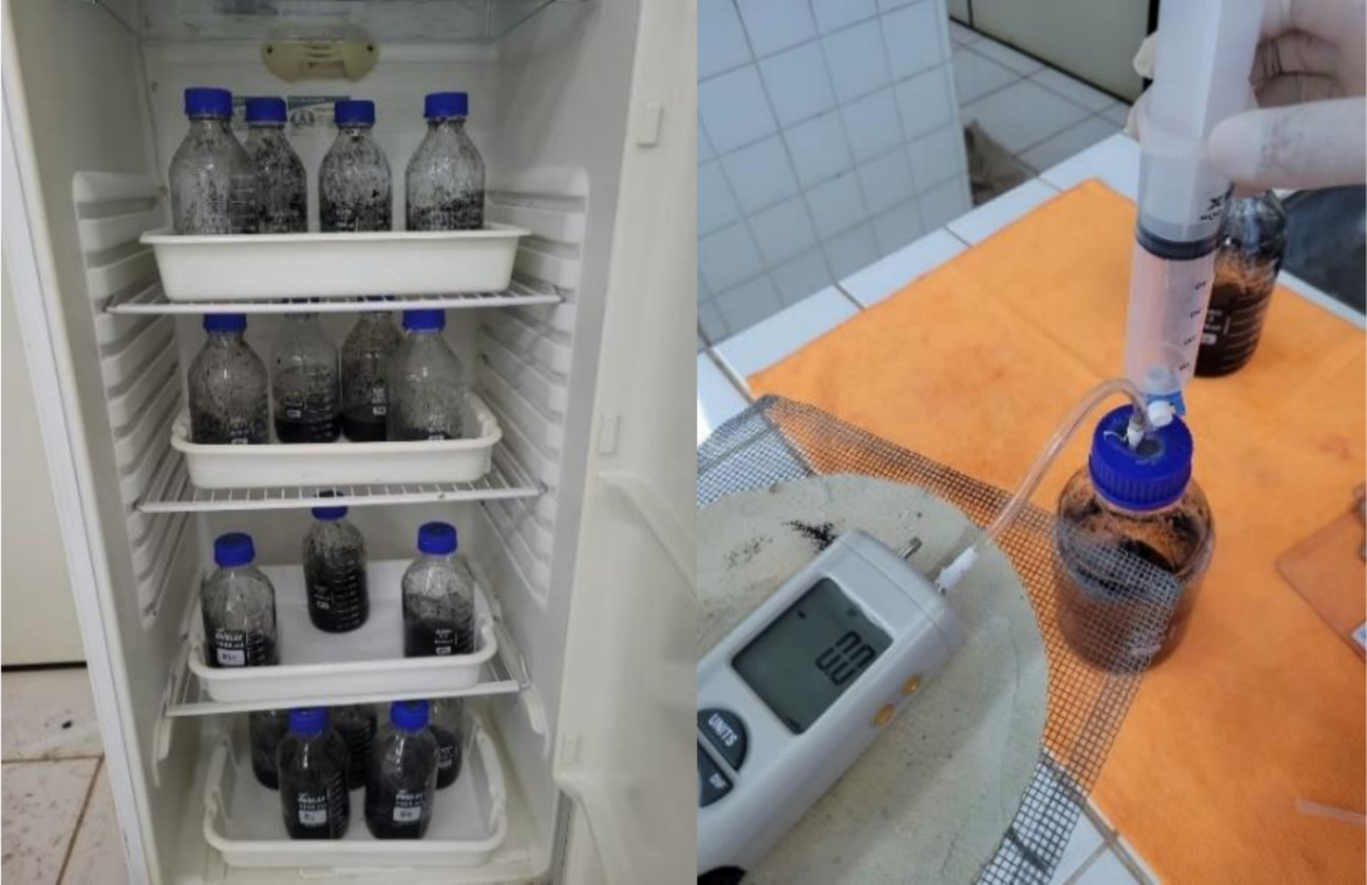
Experimental set‐up.

Borosilicate flasks were used as reactors, and the seal was made with rubber septa and screw caps. To maintain the reactors in a mesophilic temperature range of 35°C ± 2°C, a biochemical oxygen demand (BOD) chamber was used, as shown in Figure 1. Before incubation, the reactor's headspace was purged with nitrogen gas for 5 min to ensure anaerobic conditions. To correct the biogas produced from the inoculum, reactors were set up for each experiment with only anaerobic inoculum and distilled water (blank reactors). All reactors were shaken once a day for 30 s before biogas measurement. This manual mixing was adopted to simulate simplified operating conditions and reduce energy costs, although it is recognized that mass transfer may be limited compared to continuous agitation.

The daily biogas production was measured using a digital manometer (model HT‐1890; maximum pressure of 140.6 cmH_2_O; operation temperature of 0°C–50°C). A plastic syringe, equipped with a three‐way valve and a hypodermic needle (0.55 × 20 mm PrecisionGlide), was used to pierce the rubber septa without compromising the seal (Figure 1). Based on the pressure measured inside the reactors and the volume collected in the syringe, the volume was corrected considering the conditions established for the standard temperature and pressure (STP) (McNaught et al. 1997).

### 2.2 Analytical Methods

pH was measured using a Tec 3MP benchtop pH meter from the brand Tecnal before incubation, and at the end of each cycle time, the analysis of total (tCOD) and soluble COD (sCOD) (same method from tCOD, but the sample was filtered in a 0.45‐μm membrane), total Kjeldahl nitrogen (TKN), total solids (TS), fixed (TFS), volatile (TVS), dissolved (TDS) and suspended (TSS), fixed (FDS) and volatile (VDS) dissolved solids, fixed (FSS), and volatile (VSS) suspended solids were performed following APHA, AWWA, and WEF (2017). The analysis of total (TA), partial (PA), and intermediate (IA) alkalinity and volatile acids (VA) was conducted following Ripley et al. (1986) and DiLallo and Albertson (1961), respectively, before incubation and at the end of each cycle time. Moisture content (MC) and ash content (AC) determination was performed following Appiagyei Osei‐Owusu et al.'s (2023) equations, whereas glycerol content (GC) determination was performed following Vieira's (2014) proposed methodology. The values obtained in the characterization are shown in Table 1.

**TABLE 1:** Characteristics of the used substrates and inoculum.

Parameter	MWW	LL	CG	Inoculum	APHA method number
pH	7.33	8.25	—	—	4500‐H + B
TA (mgCaCO_3_/L)	221	2133	—	—	—
PA (mgCaCO_3_/L)	168	2100	—	—	—
VA (mgHAc/L)	420	620	—	—	—
tCOD (mg/L)	597.9	474.8	1,715,735.00	—	5220D
TKN (mg/L)	51.8	588.7	—	—	4500‐N_org_ C
tCOD/TKN	11.54	0.81	—	—	—
TS (mg/L)	430	2310	—	16,320	2540B
TFS (mg/L)	270	1820	—	5153.3	2540E
TVS (mg/L)	160	490	—	11166.7	2540E
TSS (mg/L)	100	150	—	11,360	2540D
FSS (mg/L)	80	100	—	3526.7	2540E
VSS (mg/L)	20	50	—	7833.3	2540E
TDS (mg/L)	330	2160	—	4960	2540C
FDS (mg/L)	190	1720	—	1626.7	2540E
VDS (mg/L)	140	440	—	3333.3	2540E
GC (%)	—	—	15.63	—	—
MC (%)	—	—	16.0	—	—
AC (%)	—	—	8.68	—	—

Abbreviations: CG: crude glycerin; LL: landfill leachate; MWW: municipal wastewater.

Although a detailed characterization of specific compounds was not performed in this study, the literature indicates that CG derived from biodiesel also contains methanol (often close to 30%), inorganic salts (such as potassium and sodium), and non–glycerol organic matter (Bansod et al. 2025; Bernat et al. 2021). These contaminants, along with recalcitrant organic matter and metals potentially present in the leachate, can exert severe inhibitory effects, which justifies investigating the dosage limits in this experimental design (Bernat et al. 2021).

Verification of the presence of organic acids in the sample at the end of the codigestion process was conducted using high‐performance liquid chromatography (HPLC) in a Shimadzu system equipped with an Aminex Column HP‐87H (300 mm × 7.8 mm Bio‐Rad), CTO‐20A oven at a temperature of 64°C, CBM‐20A controller, UV detector with SPD‐20A diode array at a wavelength of 208 nm, and a LC‐20AT pump. The mobile phase was composed of Milli‐Q (Millipore) ultrapure water acidified with 0.005 M of H_2_SO_4_ in a flux of 0.5 mL/min and an injection volume of 20 μL (Andreani et al. 2019; Penteado et al. 2013). For all organic acids analyzed, the inferior and superior detection limits were 25 and 1000 mg/L, respectively.

Prior to the composition analysis, the biogas was collected directly from the headspace, avoiding humidity and using the same procedure already described for daily biogas production measurement. Therefore, the biogas was not scrubbed or dried for analysis. Then the sample composition was determined using the gas chromatograph GC‐2014 (Shimadzu) equipped with a thermal conductivity detector (TCD) and a Carboxen 1000 (4.5 m × 2.1 mm) packed column, with temperatures for the injector and detector of 100°C and 150°C, respectively, and operated with the following specifications: argon gas as carrier gas (25 mL/min), heating of the column starting from 40°C at a rate of 20°C/min until reaching 145°C and retention times of ~12.8 min for methane and ~17.2 min for carbon dioxide.

The COD mass balance was performed considering the tCOD (input), the mass converted to methane (COD_CH4_), and the residual sCOD (output). The remaining fraction was attributed to microbial biomass, undegraded particulate solids, and any experimental losses and was termed CODparitulated + biomass. The results of the COD balance are presented in the Supporting Information.

### 2.3 Screening Design

It was adopted as a screening design, the matrix proposed by Plackett and Burman (1946). The independent variables under study were cycle time (days), pH, LL content, and CG content in the mixture. MWW was used as a base substrate to complete the reactor's working volume. Thus, the percentages of LL and CG were calculated in relation to the total working volume, with the remaining volume filled by MWW and inoculum, maintaining a constant substrate‐to‐inoculum (S/I) ratio. The S/I ratio was set at 0.4 g tCOD/gVSS for all treatments based on the results of Takeda et al. (2022), and the inoculum volume was adjusted throughout the experimental runs to maintain this ratio.

The planning presented eight runs at levels (−1) and (+1), and only two replicate runs were performed at the central point (0), totaling 10 tests as shown in Table 2. The response variables selected were the removal efficiency of sCOD and the maximum methane yield in terms of liters of methane produced per gram of tCOD_add_ (L _NCH4_/g tCOD_add_).

**TABLE 2:** Matrix of PB design with coded and uncoded variables.

Run	Cycle time (day)	pH	LL (% v/v)	CG (% v/v)	tCOD/TKN
R1	+1 (40)	−1 (7.0)	−1 (2.0)	+1 (2.0)	567.43
R2	+1 (40)	+1 (8.0)	−1 (2.0)	−1 (1.0)	286.15
R3	+1 (40)	+1 (8.0)	+1 (5.0)	−1 (1.0)	227.11
R4	−1 (20)	+1 (8.0)	+1 (5.0)	+1 (2.0)	449.62
R5	+1 (40)	−1 (7.0)	+1 (5.0)	+1 (2.0)	449.62
R6	−1 (20)	+1 (8.0)	−1 (2.0)	+1 (2.0)	567.43
R7	−1 (20)	−1 (7.0)	+1 (5.0)	−1 (1.0)	227.11
R8	−1 (20)	−1 (7.0)	−1 (2.0)	−1 (1.0)	286.15
R9	0 (30)	0 (7.5)	0 (3.5)	0 (1.5)	377.01
R10	0 (30)	0 (7.5)	0 (3.5)	0 (1.5)	377.01

Abbreviations: CG: crude glycerin; LL: landfill leachate.

The levels adopted for the study were based on research that assessed joint treatment of LL and glycerin associated with other wastewater or waste. Studies such as that by Çeçen and Aktaş (2004) found that leachate additions greater than 10% v/v would negatively impact the joint treatment with MWW, reaching values of less than 100 mg/L of sCOD removal with a leachate content of 5% v/v. Regarding CG, some research points to the need to work with smaller proportions to avoid acidification of the medium due to the accumulation of volatile acids (Astals et al. 2013; Lobato et al. 2010; Silvestre et al. 2015). Therefore, this study used as the maximum value for the proportion of leachate the value of 5% v/v. For the CG content, the maximum value was 2%, to avoid the primary inhibition mechanism, which was the overload, as well as allowing the leachate to inhibit acidification by forming a buffer system that regulated the pH (Jensen et al. 2014; Liao et al. 2014).

From the results obtained, the STATISTICA software was used to carry out an analysis of variance (ANOVA), thus identifying which fractions significantly influenced the process at a confidence interval of 90% (trust level α of 10%) and performing a nonlinear regression in two kinetic models, aiming at presenting the behavior of the treatments concerning the cumulative biogas yield. The models chosen were the modified Gompertz model and the Cone model, already used in other research to evaluate kinetic parameters of biogas production from different substances in codigestion or monodigestion or even for comparison with each other or with other models (Gadhe et al. 2013; Gulsen Akbay et al. 2021; Karki et al. 2022; Liew et al. 2021; Silva et al. 2021; Wang et al. 2020). The Gompertz model (1) and the Cone model are presented in Equations (1) and (2):

(1) $$H=P.\exp\left\{-\exp\left[\frac{R_{m}.e}{P}.\left(\lambda -t\right)+1\right]\right\}$$

(2) $$H=\frac{P}{1+{\left(\mathrm{kt}\right)}^{-n}}$$

where *H* is the cumulative methane yield (L _NCH4_/g tCOD_add_); *P* is the estimated maximum yield (L _NCH4_/g tCOD_add_); *R*_*m*_ is the maximum production rate (L _NCH4_/(g tCOD_add_.day)); *λ* is the latency phase time (day); *t* is the cycle time (day); *e* is the Euler number; *k* is the Cone kinetic constant (1/day); and *n* is a shape factor (dimensionless).

The model's adjustments were assessed through the use of the determination coefficient (*R* ^2^ and *R* ^2^ adjusted), the root mean square error (RMSE), and the normalized root mean square error (NRMSE), whereas the model's performance was assessed using the Akaike information criterion (AIC) (Coelho et al. 2020).

For model fitting under all conditions, the quasi‐Newton estimation method was employed, with a convergence criterion of 0.0001 (Cloud Software Group Inc. 2020). Initial parameter values were set to 1, except for the estimated maximum methane production, which was assigned the highest cumulative methane value observed in the experiment. It was also calculated the volatile acidity/total alkalinity (VA/TA) and intermediate alkalinity/partial alkalinity (IA/PA) ratios to serve as stability indicators of the anaerobic process (Issah and Kabera 2021; Martín‐González et al. 2013; L. S. Rodrigues et al. 2014).

## 3 Results and Discussion

### 3.1 Organic Matter Removal and Methane Yield

The physicochemical characterization of the MWW, LL, and CG used in this study is presented in Table 1. Gas chromatography analysis was used to determine the concentration of methane in biogas and present the maximum methane yield as a result. The methane content in the biogas ranged from 56.51% to 89.04% across the runs, with the remaining fraction composed primarily of carbon dioxide and traces of other gases. Table 3 presents the sCOD removal efficiency and methane yield at the end of the AcoD process. The runs R1 and R2 presented the best sCOD removals (94.3% and 94.1%, respectively), and both ran with a cycle time of 40 days. On the other hand, runs R3 and R6 presented the lowest removals (52.5% and 67.4%, respectively), with run R3 also operating for 40 days and run R6 for 20 days. Therefore, in this study, the sCOD removal range was from 52.5% to 94.3%. Kumar et al. (2023) obtained COD removal values of 60%–80% operating in sequential batch reactors with proportions of 5% and 10% in v/v of leachate to MWW, suggesting that higher leachate concentrations might be feasible under the tested conditions.

**TABLE 3:** sCOD removal and methane yield at the end of codigestion.

Run	%G —− %LL (%v/v)	sCOD input (mg/L)	sCOD output (mg/L)	Eff. (%)	Maximum methane yield (L _NCH4_/g tCOD_add_)
R1	2% LL — 2% CG	4985.4	283.6	94.3	0.285
R2	2% LL — 1% CG	5775.1	340.6	94.1	0.349
R3	5% LL — 1% CG	3405.9	906.6	73.4	0.320
R4	5% LL — 2% CG	5775.1	1512.1	73.8	0.093
R5	5% LL — 2% CG	5775.1	1591.0	72.5	0.194
R6	2% LL — 2% CG	4985.4	1626.1	67.4	0.076
R7	5% LL — 1% CG	3405.9	722.3	78.8	0.154
R8	2% LL — 1% CG	5775.1	388.9	93.3	0.245
R9*	3.5% LL — 1.5% CG	5380.3	327.4	93.9	0.197
R10*	3.5% LL — 1.5% CG	5380.3	366.9	93.2	0.189
CV%	—	—	—	0.555	2.87

Abbreviations: CG: crude glycerin; CV: coefficient of variation; Ef.: sCOD removal efficiency; LL: landfill leachate.

Table 3 shows that the maximum methane yield value was 0.349 L _NCH4_/g tCOD_add_ concerning the run R2. According to Timmerman et al. (2015), in shorter retention times, the effect of adding glycerin to increase biogas yield is reduced. Thus, it is consistent that the highest yield is for a run that operated for a longer period in the experiment and that the lowest yields are associated with runs that operated for a shorter period, such as runs R4 and R6. However, it can be verified that for runs R1 and R5, the high CG content and the cycle time of 40 days did not result in the highest maximum methane yield.

### 3.2 Plackett and Burman Screening Design Statistical Analysis

**FIGURE 2:**
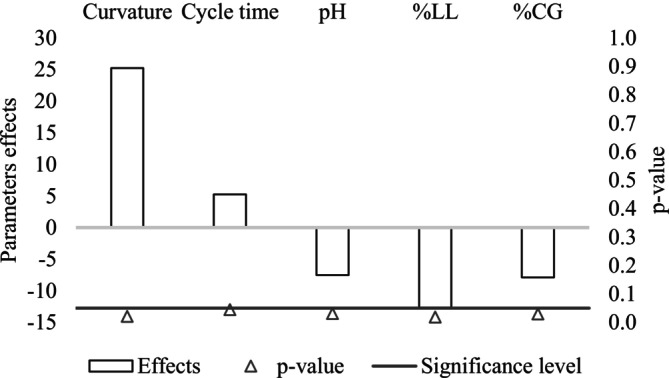
Effects and significance of independent variables in the removal of sCOD.

**FIGURE 3:**
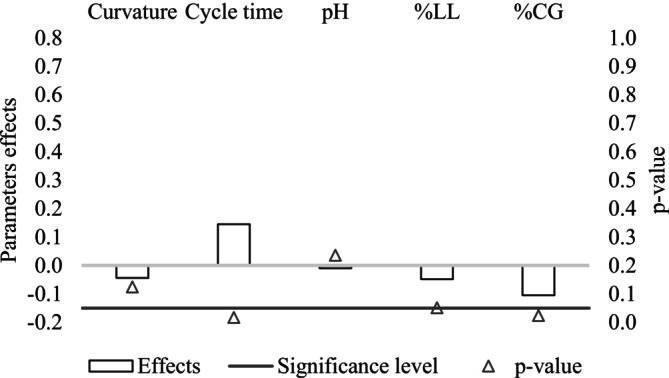
Effects and significance of independent variables on maximum methane yield.

Figures 2 and 3 present the results relative to the ANOVA carried out to verify the effects and influence of cycle time, pH, and the contents of LL and CG on the removal of sCOD and maximum methane yield. The curvature was also evaluated since the central points presented high results for the evaluated responses, which could mask the statistically significant effects of the studied variables (M. I. Rodrigues and Iemma 2014). Thus, for the removal of sCOD, the pH, LL, and CG content showed significant and negative effects, indicating that an increase in them resulted in a reduction in the removal of sCOD.

Regarding the pH variable, the value of its effect was significant for the removal of sCOD, and its effect represented a 7.53% decrease in sCOD removal when increasing the pH to 8.0. In this sense, this result shows an indication that there is no need to correct the inlet pH to values above 7.0 for the AcoD of MWW, LL, and CG. As long as the influents have a pH within the typical ranges for AcoD, there are indications that this process would occur without inhibition related to this variable (Cioabla et al. 2012).

In this study, ammonia concentrations in the samples were not measured; however, it is possible that the negative effect of the pH increase from 7 to 8 was due to partial inhibition by free ammonia, particularly considering that the conditions with removal efficiencies closer to 70% (runs R3, R4, and R6) were those that received the highest proportion of LL (Bonk et al. 2018; Jiang et al. 2019; Yang et al. 2025). Moreover, this pH value lies outside the optimal range of 6.8–7.2 for proper methanogenic activity (Gonde et al. 2023).

CG and LL content had negative effects on sCOD removal. An increase from 1.00% to 2.00% in CG content contributed to a decrease of 7.89% in sCOD removal, whereas an increase from 2.00% to 5.00% in LL content caused a decrease of 12.65% in this response variable. This states that working with lower limits is a better option and is in accordance with the literature (Astals et al. 2013; Çeçen and Aktaş 2004; Lobato et al. 2010; Silvestre et al. 2015).

Regarding maximum methane yield, increasing CG content from 1.00% to 2.00% caused an average decrease in maximum methane yield of 0.105 L _NCH4_/g tCOD_add_, as shown in Figure 3. When mixing glycerin with different wastewaters in batch‐operated reactors, glycerin content values close to 2% (v/v) have appeared as the most appropriate to increase biogas production, according to the literature (Chou and Su 2019; Takeda et al. 2022). Furthermore, increasing glycerin concentration, according to Bernat et al. (2021), can also reduce methanogenesis due to the increase in the COD/TKN ratio, especially if nitrogen is not available in the form of ammonia, which leads to an increase in the ammonification rate.

In the case of cycle time, the effect was positive and significant for both response variables. When an increase from 20 to 40 days was performed, there was an average increase in maximum methane yield of 0.145 L _NCH4_/g tCOD_add_, which explains the higher performance observed under condition R3 compared with R7. For sCOD removal, the average increase was 5.25%.

Mahmoodi‐Eshkaftaki et al. (2017) worked with a 40‐day digestion period and found that between days 30 and 35, the maximum methane yield was obtained for the AcoD of a mixture containing cow manure, municipal waste, and kitchen waste. Takeda et al. (2022) found that when mixing CG and LL, the optimum biogas production is obtained in 33 days. These findings show that even with the statistically significant effect of the cycle time, the gain in methane yield was practically low in 40 days.

However, in this study, the gain in methane yield for 40 days of cycle time represented 41% of the theoretical yield (0.350 L _NCH4_/g tCOD_add_). This shows that a significant part of the methane production is concentrated after 30 days, but the situation is different for sCOD removal.

### 3.3 Process Stability Indicators and Organic Acids

**FIGURE 4:**
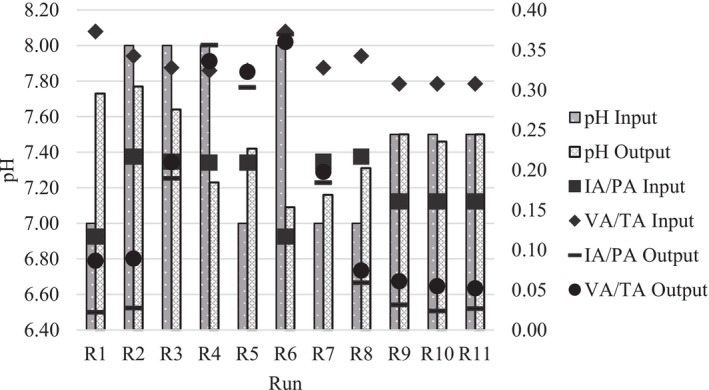
Process stability indicators.

About process stability, Figure 4 presents the values of the ratios considered as indicators. The IA/PA input values for all runs were below 0.25, with the lowest value being 0.117 for run R1. The IA/PA output values were below 0.1 for runs R1, R2, R8, R9, and R10, whereas runs R3, R4, R5, and R6 presented IA/PA output values of 0.190, 0.356, 0.303, and 0.370, respectively. Given that the IA/PA ratios below 0.3 indicate stable performance of an anaerobic reactor, the values exceeding this threshold observed under runs R4 and R6 suggest instability, which negatively impacted methane yield, resulting in the lowest values for this response (Martín‐González et al. 2013).

When analyzing the VA/TA ratio, the input values were between 0.3 and 0.4, whereas the output values had a greater variation, resulting in values below 0.10 for runs R1, R2, R8, R9, and R10. For runs R3 and R7, the VA/TA ratio resulted in 0.210 and 0.197, and for runs R4, R5, and R6, it resulted in 0.336, 0.323, and 0.360. Issah and Kabera (2021) found that values of 0.10–0.35 for the VA/TA ratio led to a good process stability with adequate removal of organic matter; thus, it is noted that only the run R6 exceeded this range, which indicates the occurrence of methane production inhibition under this condition.

When it comes to the presence of organic acids, the results of the liquid chromatography analysis are shown in Table 4, where the undetected values were below the minimum detection of the method used. These acids are formed in anaerobic digestion and used by methanogenic microorganisms to produce methane (Bian et al. 2026; Lin et al. 1986). Therefore, their presence indicates the activity of these microorganisms. Some authors suggest using the ratio between the concentration of propionic acid and acetic acid (P/A) as an indicator of system overload and a harbinger of the increased CO_2_ concentration in biogas (Marchaim and Krause 1993). Thus, the last column of Table 4 presents this ratio and, notably, that runs R3, R4, and R6 presented the highest ratios for P/A, corroborating previous results related to process stability and proximity to IA/PA and VA/TA with limit values for instability.

**TABLE 4:** Organic acids in the sample after codigestion.

Run	Lactic acid (mg/L)	Formic acid (mg/L)	Acetic acid (mg/L)	Propionic acid (mg/L)	Butyric acid (mg/L)	Propionic/acetic ratio
R1	< 25	< 25	44.40	< 25	69.51	—
R2	35.60	< 25	29.78	< 25	410.3	—
R3	35.30	257.8	38.77	203.9	790.0	5.260
R4	36.87	< 25	53.24	207.2	192.2	3.893
R5	42.04	< 25	490.4	201.7	594.9	0.4114
R6	34.85	29.15	54.36	230.9	483.6	4.247
R7	< 25	30.47	86.58	45.48	522.2	0.5253
R8	34.32	28.91	32.57	30.21	400.1	0.9274
R9	33.08	33.19	28.64	< 25	92.85	—
R10	35.27	28.53	29.11	< 25	142.1	—

Because of differences in COD loading, the reactors could present some issues related to propionic and butyric acid, as mentioned by Aquino and Chernicharo (2005). However, the highest initial loading was applied for the runs R9 and R10, but no propionic acid was detected in the effluent, and the butyric concentration detected was low in comparison with the other runs. This suggests that, for the selected levels, the contents of substrates have a more significant role in the formation of organic compounds less reduced than acetic acid.

Studies report that the anaerobic digestion of glycerol tends to result in higher propionic and butyric acid formation compared with acetic acid (Barbirato et al. 1997; Li et al. 2022; Varrone et al. 2017). These compounds were found in higher concentrations under the evaluated conditions, requiring the combined activity of acetogenic, acetoclastic methanogenic, and hydrogenotrophic methanogenic microorganisms for methane production (Aquino and Chernicharo 2005). This contributed to the lower yields observed in conditions R4 and R6, where the energy generated from conversion may have been diverted toward microbial growth.

### 3.4 Kinetic Models Adjustment to Methane Yield Data

Table 5 presents the values obtained from the nonlinear regression performed in the STATISTICA software to adjust the methane yield data to the modified Gompertz and Cone models. From this table, the values of *R* ^2^ and *R* ^2^ adjusted were below 0.9 only for the adjustment of data associated with runs R3 and R5, demonstrating that both models are adequate to describe the kinetics of methane production in the experiments. However, the modified Gompertz model appears to be the most suitable for describing the codigestion behavior of MWW, LL, and CG when it is observed for its adjustment; the lowest AIC values were obtained. When comparing different models, Karki et al. (2022) also found that the modified Gompertz model proved to be more suitable than the Cone model for codigestion, but that for the same mixture, good adjustments were obtained in different models.

**TABLE 5:** Kinetic analysis results for the two models compared.

	R1	R2	R3	R4	R5	R6	R7	R8	R9	R10
Experimental value										
*P* (L _NCH4_/g tCOD_add_)	0.285	0.349	0.320	0.0935	0.194	0.0759	0.154	0.245	0.197	0.189
Modified Gompertz										
*P* (L _NCH4_/g tCOD_add_)	0.290	0.410	0.41	0.0889	0.60	0.072	0.213	0.278	0.205	0.194
*R*_*m*_ (L _NCH4_/g tCOD_add_.day)	0.0165	0.0135	0.01	0.01	0.0038	0.0108	0.0077	0.0166	0.0124	0.0114
*λ* (day)	1.473	4.728	−0.1	−0.8238	0.2933	0.2021	−0.6542	1.5948	1.7310	1.3384
*R* ^2^	0.9973	0.9891	0.9019	0.9768	0.8575	0.9847	0.9766	0.9975	0.9989	0.9982
*R* ^2^ adjusted	0.9971	0.9885	0.8967	0.9742	0.8500	0.9830	0.9740	0.9972	0.9988	0.9981
RMSE	0.0048	0.0127	0.0260	0.0040	0.0158	0.0028	0.0068	0.0040	0.0023	0.0026
NRMSE	1.782	3.714	8.355	4.780	8.451	4.067	4.623	1.696	1.163	1.433
AIC	−418.7	−341.8	−284.3	−432.1	−321.5	−226.9	−191.3	−212.6	−357.8	−348.2
Cone										
*P* (L _NCH4_/g tCOD_add_)	0.315	0.489	0.675	0.116	1.88	0.0780	2.41	0.401	0.235	0.228
*k* (1/day)	0.0932	0.0431	0.018970	0.1349	0.0010	0.2730	0.0022	0.0671	0.0895	0.0877
*n*	2.001	2.065	1.588	1.1988	0.7564	1.8021	0.8737	1.5842	1.9082	1.7621
*R* ^2^	0.9909	0.9825	0.8966	0.9905	0.8366	0.9854	0.9906	0.9959	0.9976	0.9985
*R* ^2^ adjusted	0.9904	0.9815	0.8910	0.9894	0.8278	0.9837	0.9895	0.9954	0.9974	0.9984
RMSE	0.0084	0.0158	0.0262	0.0023	0.0162	0.0023	0.0040	0.0049	0.0032	0.0023
NRMSE	3.082	4.618	8.417	2.7063	8.6662	3.3760	2.5950	2.0827	1.6260	1.2386
AIC	−365.3	−316.1	−283.7	−222.4	−311.2	−222.1	−201.2	−193.6	−326.1	−478.5

Abbreviation: AIC: Akaike information criterion.

The negative lag phases observed for runs R3, R4, and R7 indicate that the microorganisms did not require an adaptation period under these conditions, which highlights the advantage of using inoculum in the experiment. Furthermore, these combinations were capable of providing readily accessible organic matter (Polastri et al. 2024).

**FIGURE 5:**
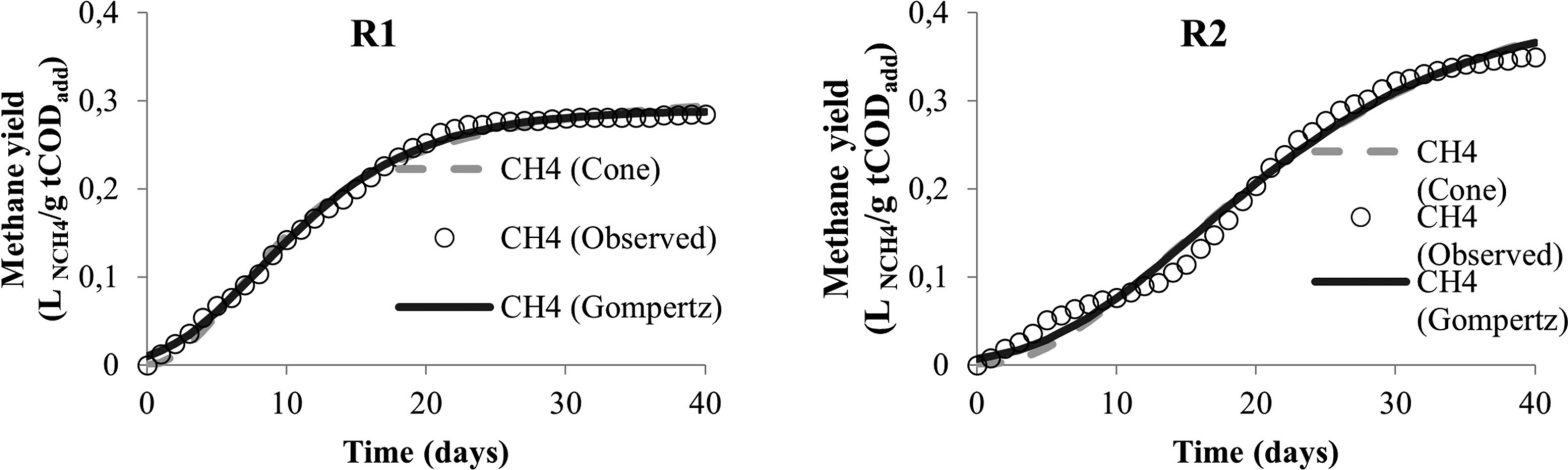
Model fit for R1 and R2 data.

**FIGURE 6:**
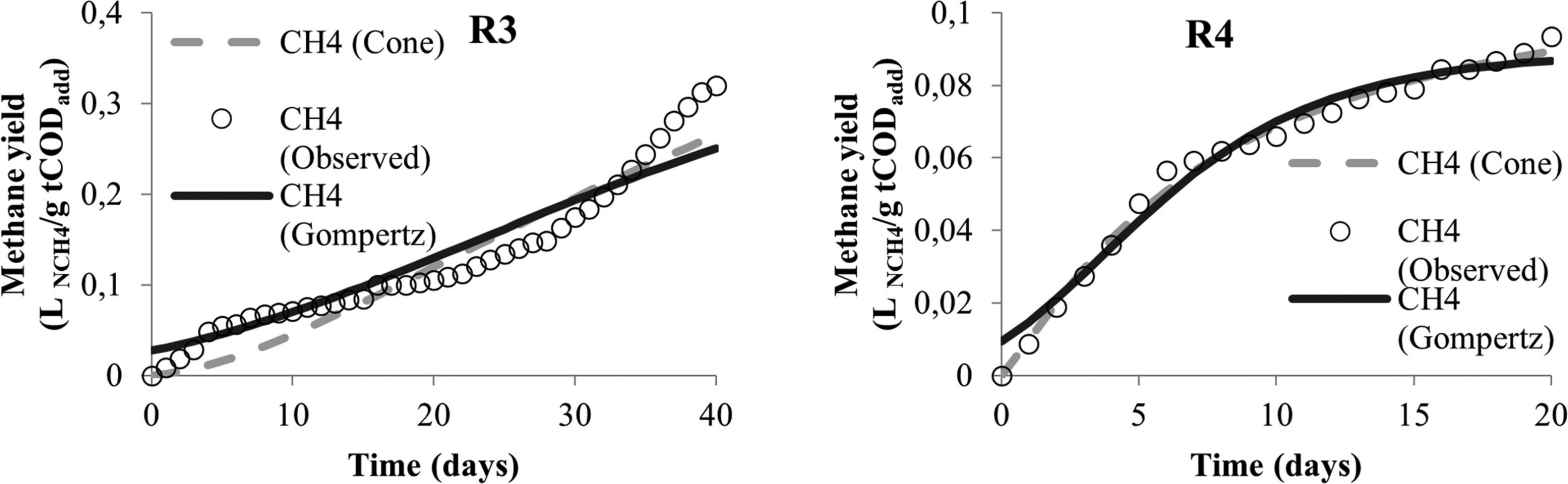
Model fit for R3 and R4 data.

**FIGURE 7:**
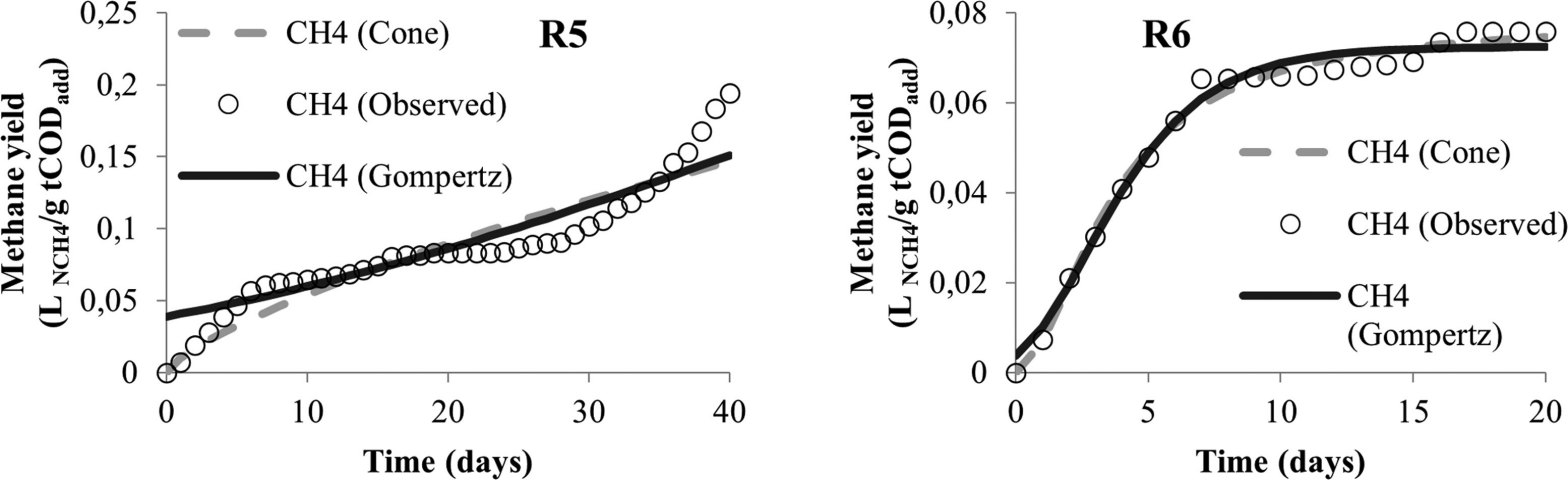
Model fit for R5 and R6 data.

**FIGURE 8:**
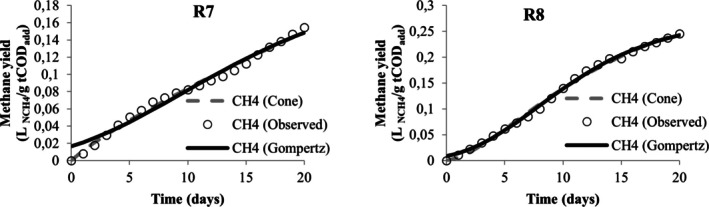
Model fit for R7 and R8 data.

**FIGURE 9:**
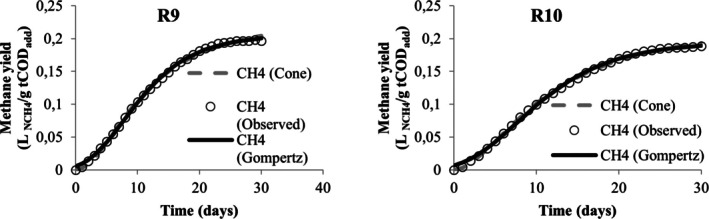
Model fit for R9 and R10 data.

Visually, the cumulative production curves for runs R1, R4, R5, R6, R7, R8, R9, and R10 resemble a reverse L‐shape curve (Figures 5, 6, 7, 8, and 9), which is a common type of curve when there is a high initial production, which is linked to the use of organic waste that has simple organic matter and is easily hydrolysable into soluble compounds such as glycerol, for example (Ware and Power 2017). Compared with the curves that Ware and Power (2017) obtained for the anaerobic digestion of soft offals, the authors discuss that the most similar form to an elongated S‐shape is associated with the low presence of fats in the substrate. In this study, the curves of runs R2, R3, R7, and R8 (Figures 5, 6, and 8) also presented a shape visually similar to an elongated S‐shape and are the runs with the lowest CG content.

According to Filer et al. (2019), the theoretical value for methane yield for standard substrates is 0.350 L _NCH4_/g COD. Comparing the experimental and the estimated yield values, the run R2 closely approached the theoretical value. The runs R4 and R6 presented the lowest values in the experiment and the modeling. Both received the highest CG concentration, and the effect on maximum methane yield was already discussed in Section 3.2.

In the Supporting Information, the quality of the fit was evaluated with respect to the confidence intervals and standard errors of the estimated parameters. Parameters with narrow confidence intervals indicated high precision in the estimate, while nonsignificant parameters (*p* > 0.05) were treated as process trends.

Also in the Supporting Information, for conditions where the model estimated a negative *λ* (e.g., runs R4 and R7), the *p*‐values associated with this parameter were not significant (*p* > 0.05). Statistically, this indicates that the lag time is not different from zero. Biologically, this confirms that the adaptation of the inoculum to the substrate was immediate, without a perceptible lag phase. Therefore, in these cases, *λ* ≈ 0 can be considered, indicating a high affinity of the biomass with the mixture (Polastri et al. 2024).

## 4 Conclusion

In this study, the codigestion of MWW, LL, and CG showed instances of inhibition due to the accumulation of organic acids, imbalances in IA/PA and VA/TA ratios, and possibly partial inhibition by free ammonia. Condition R2, which contained 2% LL and 1% CG, exhibited the highest methane yield in the experiment (0.349 L _NCH4_/g tCOD_add_), closely approaching the theoretical value (0.350 L _NCH4_/g tCOD_add_), with an sCOD removal efficiency of 94.1%. All the variables selected were statistically significant; however, the cycle time had a limited impact on methane yield (0.145 L _NCH4_/g tCOD_add_), and CG content contributed to higher COD/TKN ratios. Under these conditions, further testing is necessary to determine whether these proportions would maintain the same efficiency when subjected to other variables, such as different feeding regimes. Nonetheless, in the Brazilian context, the anaerobic cotreatment of LL in water resource recovery facilities with the addition of 1% CG is feasible. Considering the national incentive for biodiesel production through the National Biofuels Policy (RenovaBio), the surplus CG generated could be harnessed for methane production in WWTPs.

## Author Contributions

**Gustavo Henrique Pedroso:** investigation, writing – original draft, writing – review and editing, formal analysis, methodology, data curation. **Jackeline Tatiane Gotardo:** conceptualization, writing – review and editing, validation, supervision, project administration.

## Funding

This work was supported by the Coordenação de Aperfeiçoamento de Pessoal de Nível Superior.

## Conflicts of Interest

The authors declare no conflicts of interest.

## Statement of Industrial Relevance

This research is mainly related to the treatment of MWW promoted by anaerobic biological means. Therefore, the present study suggests that adding LL and CG can improve treatment efficiency and promote energy self‐sufficiency of the process through the use of biogas produced by the digestion of the combined substrates.

## Statement of Novelty

Few studies have investigated the anaerobic codigestion of MWW associated with LL and CG. Therefore, this research contributes to elucidating the feasibility of this type of approach and the advantages associated with it on a bench scale.

## Supporting information


**Data S1:** Supporting information.

## Data Availability

The data that support the findings of this study are available from the corresponding author upon reasonable request.
