# Supplementary material for: Anaerobic Codigestion of Municipal Wastewater, Landfill Leachate, and Crude Glycerin: Process Stability and Methane Yield Assessment Using a Screening Design
Source: Water Environ Res. 2026 Feb 2;98(2):e70285. doi: 10.1002/wer.70285 (PMC12865142; doi:10.1002/wer.70285)
Supplement: Supplementary file 1 — Data S1: Supporting information. [file WER-98-e70285-s001.docx]

**ANAEROBIC CO-DIGESTION OF MUNICIPAL WASTEWATER, LANDFILL LEACHATE AND CRUDE GLYCERIN: PROCESS STABILITY AND METHANE YIELD ASSESSMENT USING A SCREENING DESIGN**

Gustavo Henrique Pedroso^1*^, Jackeline Tatiane Gotardo^1^

^1^ Postgraduate Program in Agricultural Engineering (UNIOESTE/CASCAVEL/CCET/PGEAGRI), Western Paraná State University (UNIOESTE), Science Technology Center, Universitária St. 2069, Cascavel, Paraná, Brazil.

*Corresponding author: [gustavopedroso.contato@gmail.com](mailto:gustavopedroso.contato@gmail.com)

**VOLUMETRIC BIOGAS PRODUCTION AND AVERAGE BIOGAS COMPOSITION per RUN**

| **Run** | **Accumulated biogas production (L)** | **Average CH_4_ Content in biogas (%)** | **Average CO_2_ Content in biogas (%)** |
| --- | --- | --- | --- |
| R1 | 1.027 | 72.27 | 27.73 |
| R2 | 0.974 | 70.19 | 29.81 |
| R3 | 0.905 | 63.86 | 36.14 |
| R4 | 0.299 | 62.13 | 37.87 |
| R5 | 0.624 | 62.39 | 37.61 |
| R6 | 0.274 | 59.12 | 40.88 |
| R7 | 0.437 | 66.07 | 33.93 |
| R8 | 0.685 | 70.00 | 30.00 |
| R9 | 1.061 | 76.16 | 23.84 |
| R10 | 1.003 | 76.16 | 23.84 |

**COD BALANCE PER RUN**

| **Run** | **tCOD**  **(input)**  **(g)** | **COD_CH4_**  **(converted)**  **(g)^a^** | **sCOD**  **(output)**  **(g)** | **CODparticulated + biomass**  **(g)^b^** | **Error (%)** |
| --- | --- | --- | --- | --- | --- |
| R1 | 3608.39 | 2934.80 | 205.23 | 468.36 | 0 |
| R2 | 2790.06 | 2783.88 | 164.54 | 0.00 | 5.68 |
| R3 | 2829.54 | 2586.42 | 753.17 | 0.00 | 18,02 |
| R4 | 3197.06 | 854.05 | 837.06 | 1505.95 | 0 |
| R5 | 3197.06 | 1774.87 | 880.78 | 541.41 | 0 |
| R6 | 3608.39 | 782.16 | 1176.98 | 1649.25 | 0 |
| R7 | 2829.54 | 1248.09 | 600.07 | 981.37 | 0 |
| R8 | 2790.06 | 1956.21 | 187.86 | 645.99 | 0 |
| R9 | 5317.26 | 2985.97 | 323.59 | 2007.69 | 0 |
| R10 | 5317.26 | 2867.28 | 362.62 | 2087.36 | 0 |

^a^Calculated based on the volume of methane at STP.

^b^Calculated by the difference between the input and the measured outputs (Methane + Soluble), assuming retention in biomass and non-hydrolyzed solids.

**MODIFIED-GOMPERTZ MODEL**

**STATISTICS OF ESTIMATED PARAMETERS**

| R1 | | | |
| --- | --- | --- | --- |
|  | P | Rm | λ |
| Estimate | 0.2897 | 0.01654 | 1.473099 |
| Standard Error | 0.0016 | 0.00032 | 0.175564 |
| -95% CL | 0.2865 | 0.01588 | 1.117373 |
| +95% CL | 0.2928 | 0.01719 | 1.828825 |
| p-value | <0.05 | <0.05 | <0.05 |
| R2 | | | |
|  | P | Rm | λ |
| Estimate | 0.41008 | 0.01355 | 4.728009 |
| Standard Error | 0.01308 | 0.00053 | 0.514092 |
| -95% CL | 0.38358 | 0.01247 | 3.686359 |
| +95% CL | 0.43657 | 0.01463 | 5.769659 |
| p-value | <0.05 | <0.05 | <0.05 |
| R3 | | | |
|  | P | Rm | λ |
| Estimate | 0.408174 | 0.00643 | -0.10066 |
| Standard Error | 0.049366 | 0.00056 | 2.19766 |
| -95% CL | 0.308150 | 0.00530 | -4.55355 |
| +95% CL | 0.508198 | 0.00756 | 4.35223 |
| p-value | <0.05 | <0.05 | 0.96371 |
| R4 | | | |
|  | P | Rm | λ |
| Estimate | 0.08891 | 0.00734 | -0.82380 |
| Standard Error | 0.00282 | 0.00066 | 0.51201 |
| -95% CL | 0.08297 | 0.00594 | -1.90405 |
| +95% CL | 0.09486 | 0.00874 | 0.25644 |
| p-value | <0.05 | <0.05 | 0.12604 |
| R5 | | | |
|  | P | Rm | λ |
| Estimate | 0.596590 | 0.003772 | 0.2933 |
| Standard Error | 0.747494 | 0.002588 | 27.3754 |
| -95% CL | -0.917977 | -0.001473 | -55.1745 |
| +95% CL | 2.111157 | 0.009016 | 55.7611 |
| p-value | 0.429894 | 0.153493 | 0.9915 |
| R6 | | | |
|  | P | Rm | λ |
| Estimate | 0.07244 | 0.01076 | 0.202081 |
| Standard Error | 0.00107 | 0.00089 | 0.288221 |
| -95% CL | 0.07019 | 0.00887 | -0.406012 |
| +95% CL | 0.07469 | 0.01265 | 0.810173 |
| p-value | <0.05 | <0.05 | 0.492707 |
| R7 | | | |
|  | P | Rm | λ |
| Estimate | 0.212900 | 0.00766 | -0.65417 |
| Standard Error | 0.031436 | 0.00038 | 0.50425 |
| -95% CL | 0.146575 | 0.00687 | -1.71804 |
| +95% CL | 0.279225 | 0.00846 | 0.40969 |
| p-value | <0.05 | <0.05 | 0.21185 |
| R8 | | | |
|  | P | Rm | λ |
| Estimate | 0.27789 | 0.01662 | 1.594850 |
| Standard Error | 0.00626 | 0.00039 | 0.162840 |
| -95% CL | 0.26469 | 0.01579 | 1.251286 |
| +95% CL | 0.29110 | 0.01745 | 1.938413 |
| p-value | <0.05 | <0.05 | <0.05 |
| R9 | | | |
|  | P | Rm | λ |
| Estimate | 0.2054 | 0.01243 | 1.73096 |
| Standard Error | 0.0012 | 0.00017 | 0.10923 |
| -95% CL | 0.2031 | 0.01208 | 1.50685 |
| +95% CL | 0.2078 | 0.01279 | 1.95508 |
| p-value | <0.05 | <0.05 | <0.05 |
| R10 | | | |
|  | P | Rm | λ |
| Estimate | 0.1945 | 0.01140 | 1.338426 |
| Standard Error | 0.0014 | 0.00020 | 0.137871 |
| -95% CL | 0.1916 | 0.01099 | 1.055538 |
| +95% CL | 0.1973 | 0.01180 | 1.621313 |
| p-value | <0.05 | <0.05 | <0.05 |

**CONE MODEL**

**STATISTICS OF ESTIMATED PARAMETERS**

| R1 | | | |
| --- | --- | --- | --- |
|  | P | k | n |
| Estimate | 0.31475 | 0.09318 | 2.00074 |
| Standard Error | 0.00613 | 0.00261 | 0.10855 |
| t | 51.34326 | 35.70256 | 18.43152 |
| -95% CL | 0.30233 | 0.08789 | 1.78080 |
| +95% CL | 0.32717 | 0.09847 | 2.22068 |
| p-value | <0.05 | <0.05 | <0.05 |
| R2 | | | |
|  | P | k | n |
| Estimate | 0.48878 | 0.043085 | 2.064521 |
| Standard Error | 0.04811 | 0.004679 | 0.228159 |
| t | 10.15887 | 9.207777 | 9.048610 |
| -95% CL | 0.39129 | 0.033604 | 1.602227 |
| +95% CL | 0.58627 | 0.052566 | 2.526815 |
| p-value | <0.05 | <0.05 | <0.05 |
| R3 | | | |
|  | P | k | n |
| Estimate | 0.674545 | 0.018970 | 1.587713 |
| Standard Error | 0.090653 | 0.003068 | 0.189430 |
| t | 7.440964 | 6.183245 | 8.381510 |
| -95% CL | 0.490865 | 0.012754 | 1.203890 |
| +95% CL | 0.858225 | 0.025186 | 1.971535 |
| p-value | <0.05 | <0.05 | <0.05 |
| R4 | | | |
|  | P | k | n |
| Estimate | 0.11649 | 0.134858 | 1.19878 |
| Standard Error | 0.00958 | 0.021352 | 0.11881 |
| t | 12.16562 | 6.316037 | 10.08947 |
| -95% CL | 0.09629 | 0.089810 | 0.94810 |
| +95% CL | 0.13669 | 0.179906 | 1.44946 |
| p-value | <0.05 | <0.05 | <0.05 |
| R5 | | | |
|  | P | k | n |
| Estimate | 1.882029 | 0.000951 | 0.756430 |
| Standard Error | 1.298236 | 0.001117 | 0.088011 |
| t | 1.449681 | 0.851245 | <0.05 |
| -95% CL | -0.748448 | -0.001312 | 0.578102 |
| +95% CL | 4.512505 | 0.003213 | 0.934758 |
| p-value | 0.155571 | 0.400109 | <0.05 |
| R6 | | | |
|  | P | k | n |
| Estimate | 0.07799 | 0.27298 | 1.80210 |
| Standard Error | 0.00200 | 0.01277 | 0.16256 |
| t | 38.98201 | 21.37041 | 11.08565 |
| -95% CL | 0.07377 | 0.24603 | 1.45913 |
| +95% CL | 0.08221 | 0.29993 | 2.14508 |
| p-value | <0.05 | <0.05 | <0.05 |
| R7 | | | |
|  | P | k | n |
| Estimate | 2.404892 | 0.002226 | 0.87365 |
| Standard Error | 0.959907 | 0.001196 | 0.03115 |
| t | 2.505339 | 1.860528 | 28.05092 |
| -95% CL | 0.379666 | -0.000298 | 0.80794 |
| +95% CL | 4.430117 | 0.004749 | 0.93936 |
| p-value | <0.05 | 0.080200 | <0.05 |
| R8 | | | |
|  | P | k | n |
| Estimate | 0.401108 | 0.067135 | 1.58420 |
| Standard Error | 0.055754 | 0.012187 | 0.14887 |
| t | 7.194194 | 5.508694 | 10.64180 |
| -95% CL | 0.283477 | 0.041423 | 1.27012 |
| +95% CL | 0.518740 | 0.092848 | 1.89828 |
| p-value | <0.05 | <0.05 | <0.05 |
| R9 | | | |
|  | P | k | n |
| Estimate | 0.23514 | 0.08951 | 1.90822 |
| Standard Error | 0.00474 | 0.00241 | 0.06877 |
| t | 49.60967 | 37.16865 | 27.74674 |
| -95% CL | 0.22541 | 0.08457 | 1.76711 |
| +95% CL | 0.24486 | 0.09445 | 2.04934 |
| p-value | <0.05 | <0.05 | <0.05 |
| R10 | | | |
|  | P | k | n |
| Estimate | 0.23514 | 0.08951 | 1.90822 |
| Standard Error | 0.00474 | 0.00241 | 0.06877 |
| t | 49.60967 | 37.16865 | 27.74674 |
| -95% CL | 0.22541 | 0.08457 | 1.76711 |
| +95% CL | 0.24486 | 0.09445 | 2.04934 |
| p-value | <0.05 | <0.05 | <0.05 |
